# Identification of CMTM7 as a Transmembrane Linker of BLNK and the B-Cell Receptor

**DOI:** 10.1371/journal.pone.0031829

**Published:** 2012-02-21

**Authors:** Atsuko Miyazaki, Satomi Yogosawa, Akikazu Murakami, Daisuke Kitamura

**Affiliations:** 1 Division of Molecular Biology Laboratory, Research Institute for Biological Sciences (RIBS), Tokyo University of Science, Noda, Chiba, Japan; 2 Division of Azuma Laboratory, Research Institute for Biological Sciences (RIBS), Tokyo University of Science, Noda, Chiba, Japan; Universidade Federal do Rio de Janeiro, Brazil

## Abstract

BLNK is a pivotal adaptor protein in the signal transduction pathway from the IgM class B-cell receptor. BLNK is phosphorylated by Syk and binds various signaling intermediates, leading to cellular events including MAP-kinase activation, culminating in cellular activation. It remains unclear how BLNK is initially recruited to the surface IgM (sIgM) complex to which Syk is also recruited. Here we show that CMTM7, a tetra-spanning membrane protein of unknown function, co-localized with clathrin and sIgM at the plasma membrane. RNA-interference-mediated knockdown of *CMTM7* expression in B cells resulted in an impairment of sIgM-ligation-induced tyrosine phosphorylation of BLNK, which was due to an impaired interaction of BLNK and Syk, and in a failure to activate JNK and ERK, but not upstream kinases such as Src-family kinases and Syk. CMTM7 was bound to BLNK in a membrane fraction, and their association was augmented after sIgM ligation. Exogenous CMTM7 or a mutant with an N-terminal deletion (ΔN), but not one with a C-terminal deletion (ΔC) that is defective in membrane localization, were able to restore BLNK-Syk binding, BLNK phosphorylation and ERK activation in the CMTM7-knockdown B cells. In addition, CMTM7 and the ΔN, but not the ΔC, were constitutively associated with sIgM, and this binding was required for BLNK recruitment to sIgM. From these data, we conclude that CMTM7 functions to link sIgM and BLNK in the plasma membrane, to recruit BLNK to the vicinity of Syk, and to initiate the BLNK-mediated signal transduction.

## Introduction

Upon ligation with antigen, B-cell antigen receptors (BCR) cluster on the cell surface, rapidly transduce signals into the cytoplasm, and are eventually internalized with bound antigen, primarily through a clathrin-mediated endocytosis pathway [1], [2]. The BCR on the membrane of naïve B cells is a complex composed of surface immunoglobulin M (sIgM) and the signal transducing subunits, Igα and Igβ. Signal transduction is initiated with the phosphorylation by Src-family kinases such as Lyn of tyrosine residues in an immunoreceptor tyrosine-based activation motif (ITAM) contained within the cytoplasmic domains of both Igα and Igβ [3]. Syk is then recruited to the ITAM phosphotyrosines, activated and subsequently phosphorylates the adaptor protein BLNK to which signaling factors such as Btk, phospholipase Cγ2 (PLCγ2), Vav and Grb2 are recruited through their SH2 or SH3 domains. Syk then phosphorylates and activates Btk and Vav, which activate PLCγ2 and Rac, respectively. The activated PLCγ2 hydrolyzes phosphatidylinositol 4,5-bisphosphate into inositol trisphosphate (IP_3_) and diacyl glycerol (DAG). IP_3_ triggers Ca^2+^ mobilization, while DAG activates Ras through RasGRP [4], [5]. Ras and Rac trigger signaling cascades eventually activating MAP-kinases such as ERK and JNK. Intracellular calcium and DAG also activate enzymes such as PKC, which initiates signaling cascades including those activating NF-κB. These biochemical events culminate in the activation of transcription factors that induce activation, proliferation and/or differentiation of B cells [6].

BLNK (also known as SLP65 or BASH) plays a crucial role in signal transduction from the BCR, especially of the IgM class, and the pre-BCR. In BLNK-deficient mice, B-cell development is markedly affected at both pre-B-cell and immature B-cell stages. The spleen contains fewer mature B cells than normal, and the B cells present respond poorly to BCR ligation-induced proliferation *in vitro* and the mice also have a defective antibody response to T-independent type-2 antigens *in vivo* [7]–[11]. BLNK-deficient B cells also have defects in BCR-triggered Ca^2+^ flux and activation of PLCγ2, ERK, JNK, p38, and NF-κB [7], [8], [12], [13]. Thus, BLNK functions as a multivalent adaptor molecule that gathers signaling intermediates to form a ‘signalosome’ beneath the sIgM complex.

In order to exert its adaptor function, BLNK must first be recruited to the BCR to be phosphorylated by Syk, which is bound there to Igα/Igβ. However, the mechanism for BLNK recruitment to the BCR remains unclear. It has been shown that, upon phosphorylation, a non-ITAM tyrosine (Y^204^ in mice) of Igα binds the C-terminal src-homology 2 (SH2) domain of BLNK, and that this contributes to BLNK phosphorylation upon BCR- or Igα-crosslinking to various extents depending on the experimental setting [14]–[18]. It has also been proposed that BLNK directly binds to Syk [19] through the BLNK SH2 domain and the C-terminal region of Syk [20], [21]. However, deletion of the SH2 domain resulted only in a modest reduction of tyrosine phosphorylation of BLNK upon BCR ligation [20], implicating an SH2 domain-independent mechanism for the interaction of BLNK with Syk. An N-terminal basic region of BLNK, and most likely a leucine zipper motif in this region, was shown to be necessary and sufficient for constitutive association of BLNK with the plasma membrane [22], although a BLNK variant lacking the leucine zipper motif could still be phosphorylated by Syk in a T-cell reconstitution system [20].

We have previously identified CMTM3 (formerly termed BNAS2) as a binding partner of BLNK [23]. CMTM3 belongs to a nine-member protein family (CKLF and CMTM1∼8) of unknown function, which have homology to a tetra-spanning transmembrane domain called MARVEL (MAL and related protein for vesicle trafficking and membrane linking) [24]. Some of the MARVEL domain-containing proteins are involved in cholesterol-rich membrane apposition events, such as biogenesis of vesicular transport carriers or tight junction regulation, but the functions of others remain unknown [25]. Genes encoding this family, except for *CMTM5*, cluster at two loci on different chromosomes; *CKLF* and *CMTM1∼4* on chromosome 8 in mice and 16 in humans, and *CMTM6∼8* on chromosome 9 in mice and 3 in humans. The expression profile of each member shows a distinctive pattern, with CMTM3 and 7 being expressed selectively in hematopoietic cells. In the course of our study of CMTM3 function, we noted that CMTM7 is also bound with BLNK. Thus far, no reports have been published about CMTM7 except for one describing its genomic configuration [24]. Here we report that CMTM7 is crucial for BCR-induced BLNK interaction with and phosphorylation by Syk, and for activation of downstream signaling pathways to ERK and JNK. We propose that CMTM7 functions to connect sIgM and BLNK, thus facilitating formation of the BLNK-nucleated sIgM signalosome.

## Results

### CMTM7 is localized at the plasma membrane in association with clathrin and surface IgM

**Figure 1:**
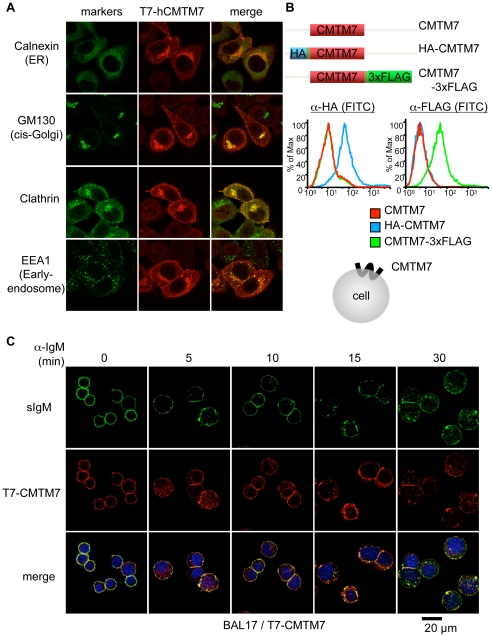
CMTM7 is localized at the plasma membrane in association with clathrin and sIgM. (A) HeLa cells stably expressing T7-hCMTM7 were fixed, permeabilized, and stained with antibodies for the indicated organelle makers (left panels) and T7 tag (middle panels) as described in the Materials and Methods section. Right panels: merged images. (B) HEK293T cells transiently expressing mouse CMTM7 tagged with HA or FLAG epitopes at the N- or C-termini, respectively, or not (schematically represented at the top), were stained with anti-HA antibody (left) or anti-FLAG antibody (right) and analyzed by flow cytometry. The histograms indicate fluorescence intensities for non-tagged (red lines), and HA- (blue lines) or FLAG- (green lines) tagged CMTM7s. The predicted plasma membrane topology of CMTM7 is depicted at the bottom. (C) BAL17 cells stably expressing T7-tagged mouse CMTM7 (BAL17/T7-CMTM7) were stained with rabbit anti-T7 and biotin-anti-IgM antibodies, and incubated at 37°C for the indicated time periods. Then the cells were fixed, permeabilized, and stained with TRITC-anti-rabbit IgG and FITC-streptavidin. The fluorescence FITC (top), TRITC (middle), and their merged images (bottom) are shown.

To clarify the localization of CMTM7 in mammalian cells, we transfected HeLa cells with a T7 epitope-tagged human CMTM7 expression construct and stained the cells with anti-T7 antibody together with antibodies against various organelle markers. Confocal microscopy revealed that CMTM7 is co-localized with clathrin accumulating near the plasma membrane, presumably representing clathrin-coated pits and vesicles, in a perinuclear region partly overlapping with the Golgi apparatus, and also with some early endosomes, whereas there was no apparent co-localization with the endoplasmic reticulum (ER) marker calnexin (**Figure 1A**). To clarify the membrane topology of CMTM7, HEK293T cells were transfected with expression constructs encoding mouse CMTM7 tagged with HA or FLAG epitopes at N- or C-termini, respectively. Both epitopes could be detected with specific antibodies by flow cytometry on non-permeabilized cells (**Figure 1B**), indicating that both the N- and C-terminal moieties of CMTM7 are exposed outside of cells.

To confirm the plasma membrane localization of CMTM7 in B cells and examine possible changes in localization dynamics after ligation of surface IgM (sIgM), we made a mouse B-cell lymphoma cell line, BAL17, stably transfected with an expression construct encoding T7-tagged mouse CMTM7 (BAL17/T7-CMTM7). The intact BAL17/T7-CMTM7 cells were stained with anti-T7 and anti-IgM antibodies and cultured at 37°C to allow the ligation-mediated reorganization of sIgM and its subsequent signaling. Before the culture, CMTM7 was clearly detected at plasma membrane, co-localizing almost exactly with sIgM. Some of the CMTM7 were internalized into cytoplasm by 5 minutes after the initiation of the culture, but others remaining on the plasma membrane were concomitantly segregated with sIgM into clusters. By 15 minutes, sIgM was internalized with the surface CMTM7 and mostly co-localized with CMTM7 at vesicular structures in the cytoplasm (**Figure 1C**). The observed tight association of sIgM and CMTM7 during the active sIgM movement strongly suggests a physical interaction between the two molecules.

### CMTM7 is required for BLNK phosphorylation by Syk and for signal transduction upon sIgM ligation

**Figure 2:**
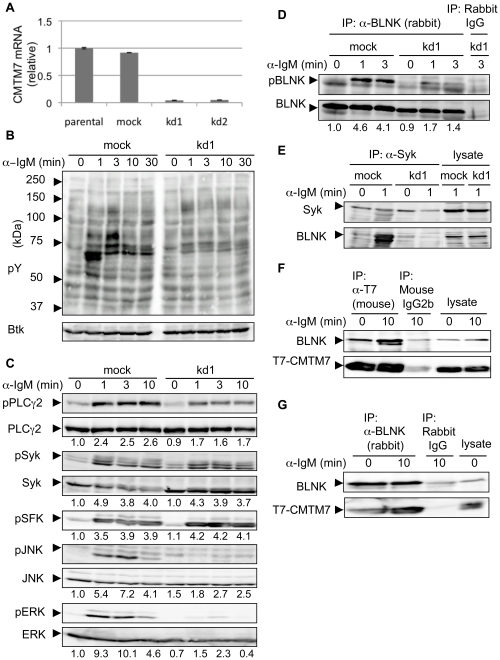
CMTM7 is required for BLNK association with Syk, its phosphorylation and signal transduction from BCR. (A) Expression levels of endogenous *CMTM7* mRNA in parental, mock-transduced, and in two independent CMTM7-knockdown BAL17 cells (kd1 and kd2) evaluated by real-time RT-PCR. (B, C, D, E) The kd1 or the mock-transduced BAL17 cells were stimulated with anti-IgM antibody for the indicated time periods. (B, C) The cell lysates were subjected to Western blot analysis with antibodies against the indicated molecules. pY: total phosphotyrosines detected by the PY20 antibody; SFK: src-family kinase. (D, E) The cell lysates were immunoprecipitated (IP) with anti-BLNK antibody or control rabbit IgG (D), or with anti-Syk antibody (E), and the precipitates and the lysates were subjected to Western blot analysis with the indicated antibodies. (F, G) BAL17/T7-CMTM7 cells were stimulated with anti-IgM for 10 min or untreated (0 min), and the cell lysates were immunoprecipitated (IP) with the indicated antibodies or species- and isotype-matched control antibodies and analyzed as in (E). (C, D) Numbers below each panel represent relative phosphorylation values of each protein (setting the value of the left-most sample in each panel as 1.0), normalized as relative to the corresponding total proteins as described in the Materials and Methods, except for pSFK which contains the undefined number of Src-family proteins.

To investigate the function of CMTM7 in B cells, we generated BAL17 transductants in which *CMTM7* gene expression was suppressed by shRNAs targeting two independent sequences (kd1 and kd2), as well as cells transduced with a control shRNA vector (mock) (**Figure 2A**). We found that BCR-ligation-induced tyrosine-phosphorylation of several proteins was attenuated in the CMTM7-knockdown cells as compared with the mock-transduced cells (**Figure 2B** **and Figure S1A**). Activation-associated phosphorylation of ERK and JNK was greatly attenuated and that of PLCγ2 was moderately, whereas that of Src-family kinases (SFK) and Syk were unaffected by CMTM7 knockdown (**Figure 2C** **and Figure S1B**). In addition, BCR-induced tyrosine-phosphorylation of BLNK was greatly attenuated in the CMTM7-knockdown cells (**Figure 2D** **and Figure S1C**), which was attributable to impaired association of BLNK with Syk (**Figure 2E** **and Figure S1D**). Moreover, reciprocal immunoprecipitation experiments demonstrated association of CMTM7 and BLNK, which was augmented after sIgM-ligation (**Figure 2F and 2G**). These data strongly suggest that, upon sIgM-ligation, CMTM7 functions to recruit BLNK to the plasma membrane in the vicinity of BCR-bound Syk and thus facilitate BLNK phosphorylation by Syk.

### The C-terminal region of CMTM7 is necessary for its membrane localization and for BLNK phosphorylation

**Figure 3:**
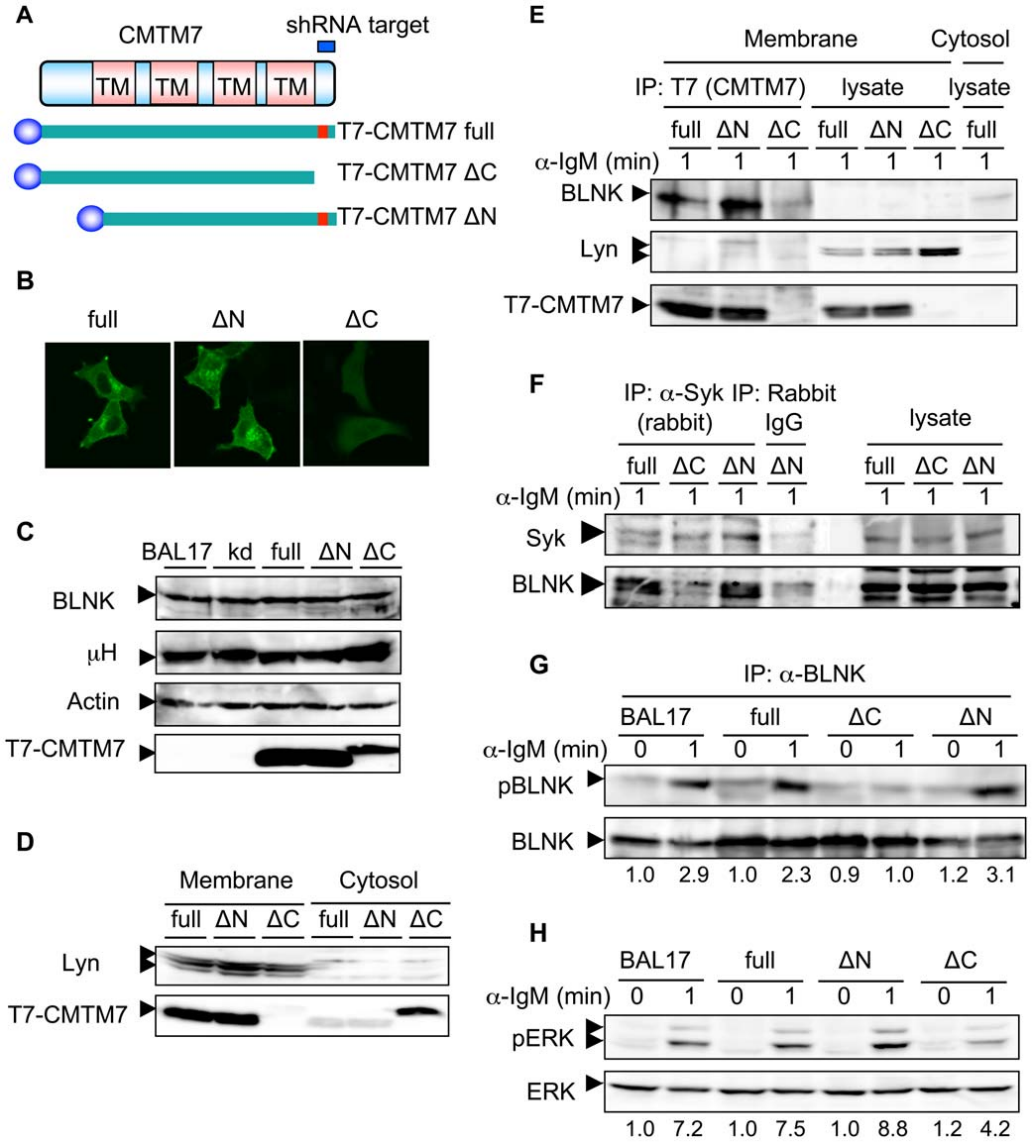
The C-terminal region of CMTM7 is necessary for its membrane localization and function. (A) Schematic representation of the mouse CMTM7 cDNA, and a T7-tagged (indicated by a blue globule) full-length version (full) or deletion mutants lacking the N-terminal (ΔN) or the C-terminal (ΔC) putative extracellular portions. Double point mutations (indicated in red) were introduced at the shRNA recognition site of the full and the ΔN CMTM7s. (B) HeLa cells transiently transfected with the full, ΔN, or ΔC CMTM7s were fixed, permeabilized, stained with anti-T7 antibody, and analyzed by confocal microscopy. (C) Western blot analysis of BAL17 cells, kd1 cells, and the latter reconstituted with the CMTM7 forms shown in (A). Antibodies against BLNK, IgM H chain (μ H), β-actin, and T7 tag (for the exogenous CMTM7s) were used for detection. (D) The reconstituted kd1 cells were fractionated into membrane and cytosol, and the lysates of both fractions were subjected to Western blot analysis using antibodies against Lyn and the T7-tag. (E) The same cells as in (D) were stimulated with anti-IgM for 1 min, and the lysates of the membrane fractions of these cells were immunoprecipitated with anti-T7 antibody [IP:T7(CMTM7)]. The precipitates and the lysates, as well as a cytosol fraction of the ‘full’ cells (the most right lane), were analyzed as in (D) with the addition of an anti-BLNK antibody. (F, G, H) BAL17 cells and the reconstituted kd1 cells were stimulated with anti-IgM antibody for the indicated time periods and the cell lysates were immunoprecipitated with the indicated antibodies (IP). The precipitates (F, G) and the lysates (F, H) were analyzed by Western blotting for the presence of BLNK and Syk (F), tyrosine-phosphorylated (pBLNK) and total BLNK (G), or activated (pERK) and total ERK (H). (G, H) Numbers below the panels represent relative phosphorylation values of each protein (setting the value of the left-most sample in each panel as 1.0), normalized as relative to the corresponding total proteins as described in the Materials and Methods.

To further investigate the mode of CMTM7 interaction, we made expression vectors that encode T7-tagged full length (full) CMTM7, and mutants lacking the N-terminal (ΔN) or the C-terminal (ΔC) putative extracellular regions. A silent mutation at the shRNA recognition site was introduced into the full and ΔN constructs to prevent their recognition and silencing by the shRNA (**Figure 3A**). When transiently expressed in HeLa cells, the full and ΔN versions of CMTM7 were localized to the plasma membrane and perinuclear regions as described above, whereas the ΔC version was diffusely localized in the cytosol and the nucleus (**Figure 3B**). We transfected these constructs into CMTM7-knockdown BAL17 (kd1) cells and made stable clones expressing the full, ΔN, or ΔC versions of CMTM7 proteins. These clones as well as the original BAL17 and parental kd1 cells expressed equivalent levels of BLNK and IgM H chain proteins (**Figure 3C**). A subcellular fractionation experiment confirmed that most of the full and ΔN CMTM7 proteins were present in the membrane fraction, whereas the ΔC was exclusively in a cytosolic fraction (**Figure 3D**). In the membrane fraction, the full and the ΔN CMTM7 were bound with BLNK but not Lyn (**Figure 3E**). BCR-induced BLNK association with Syk was restored in the kd1 cells reconstituted with the full or the ΔN, but not the ΔC, CMTM7 (**Figure 3F**), indicating that the C-terminal domain of CMTM7, or membrane localization of CMTM7, is critical for the binding of both proteins. Accordingly, BCR-induced tyrosine-phosphorylation of BLNK and ERK activation were fully restored in the kd1 cells reconstituted with the full or the ΔN, but not the ΔC, CMTM7 (**Figure 3G and 3H**).

### CMTM7 is associated with sIgM and recruits BLNK

**Figure 4:**
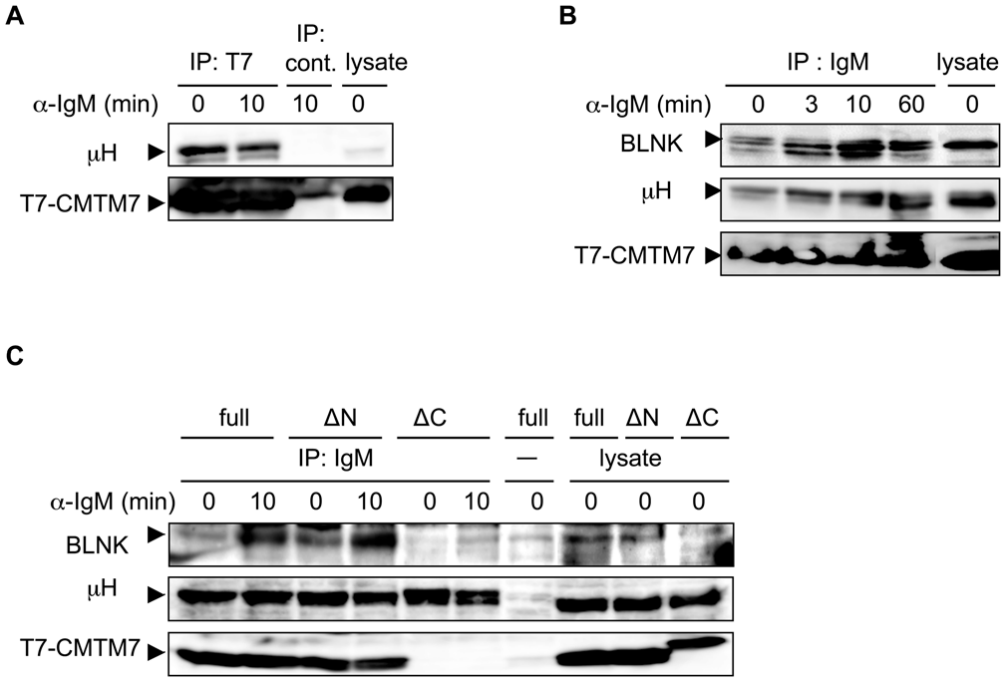
CMTM7 binds to sIgM and recruits BLNK to sIgM. (A) BAL17/T7-CMTM7 cells were stimulated with anti-IgM antibody for 10 min or untreated (0 min). Then the cell lysates were immunoprecipitated with mouse anti-T7 (IP: T7) or an isotype-matched control (IP: cont.) monoclonal antibodies. The precipitates and the lysate were analyzed by Western blot analysis. (B, C) BAL17/T7-CMTM7 cells (B) or the kd1 cells reconstituted with the indicated forms of CMTM7 (C) were treated with goat anti-IgM antibody (whole IgG) or untreated (-) and incubated at 37°C for the indicated time periods, or kept on ice (0 min). Then the cell lysates were incubated with protein G-coated beads. The bead-bound proteins (IP: IgM or -) and the lysates were analyzed by Western blot analysis using antibodies against μ H chain, T7 tag, and BLNK as indicated.

It is well known that, upon ligation of IgM, Syk is recruited to the Igα/Igβ subunits of the IgM BCR complex via binding to phosphorylated ITAMs in their cytoplasmic tails [3]. Therefore, the observed CMTM7-dependent interaction of BLNK with Syk led us to hypothesize that CMTM7 is associated with the IgM complex and thus mediates interaction of BLNK and Syk. Indeed, μH chain was co-precipitated with CMTM7 from BAL17/T7-CMTM7 cell lysates (**Figure 4A**), and CMTM7 was present in the surface IgM complex in the same cells irrespective of sIgM ligation (**Figure 4B**). Although both full and ΔN CMTM7 was co-precipitated with sIgM in the reconstituted kd1 cells, ΔC CMTM7 was not (**Figure 4C**). Furthermore, BLNK was co-precipitated with the sIgM in the BAL17/T7-CMTM7 cells (**Figure 4B**) and the kd1 cells reconstituted with the full or ΔN, but not ΔC, CMTM7 (**Figure 4C**). Therefore, BLNK recruitment to the sIgM complex is dependent on CMTM7 integrated in the plasma membrane.

## Discussion

**Figure 5:**
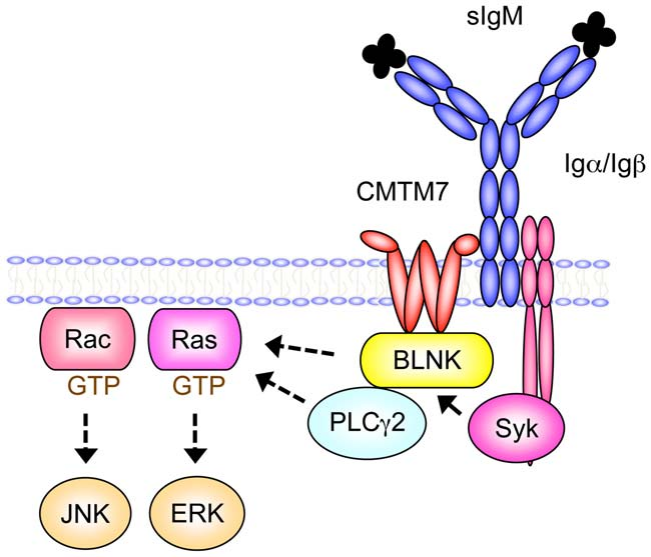
A model for the CMTM7 function in the B cell receptor complex. CMTM7 is associated with sIgM where it mediates interaction of BLNK and Syk, as well as BLNK phosphorylation by Syk, which is necessary for eventual activation of ERK and JNK, in part through PLCγ2 activation. Arrows with solid and dotted lines indicate direct and indirect interactions, respectively.

BLNK has been characterized as a multivalent adaptor protein that plays a pivotal role in signal transduction from BCR of the IgM class. It has been demonstrated that Syk is the dominant tyrosine kinase that phosphorylates BLNK and that this phosphorylation is mandatory for the BLNK adaptor function [26]. However, less is known how BLNK is initially recruited to the sIgM complex, to which Syk is already recruited and thereby activated, after ligation of the BCR. Here we have identified a previously unknown transmembrane protein CMTM7 as the missing link between BLNK and the BCR. We demonstrated that CMTM7 binds both BLNK and sIgM, and is necessary for Syk interaction with and phosphorylation of BLNK, and ultimately for activation of downstream MAP-kinases after sIgM ligation (**Figure 5**). Confocal microscopy data indicated a tight association of the two proteins in the plasma membrane as well as in the membrane of vesicles trafficking through the cytoplasm. Although the diffuse cytoplasmic localization of BLNK hampered confocal microscopic visualization of its plasma membrane recruitment, biochemical analyses clearly demonstrated that BLNK is bound with CMTM7 in the membrane fraction, and also that BLNK is associated with the sIgM complex only when CMTM7 is normally integrated in the plasma membrane. These data strongly suggest that CMTM7 is a part of sIgM complex that binds BLNK for its phosphorylation by Syk and to nucleate the ‘signalosome’.

BLNK has been shown to directly bind to Igα [14]–[16] or to Syk [20], [21] via its SH2 domain. While these interactions appear to be biologically significant, they do not appear to be essential for BLNK phosphorylation since BLNK lacking the SH2 domain could still be phosphorylated in B cells upon BCR ligation [20]. Likewise, the N-terminal leucine zipper motif of BLNK, a requisite for its plasma-membrane association [22], is not essential for BLNK phosphorylation [20]. Nevertheless, given their considerable contributions to BLNK phosphorylation, these interactions are likely involved in the process whereby BLNK is recruited to the sIgM complex and interacts with Syk, in addition to the CMTM7-BLNK interaction demonstrated in the present study. Whether these interactions represent different steps of the process leading to full BLNK phosphorylation, or are redundant mechanisms for this process, remains to be examined. Our data demonstrated that BLNK is co-precipitated with CMTM7 before sIgM ligation but that the amount of co-precipitate increased after the ligation. It appears that crosslinking of sIgM causes multimerization of the associated CMTM7, which may stabilize the binding of BLNK. BLNK SH2 domain-mediated binding to Igα and/or Syk may further stabilize the association of BLNK with the sIgM complex.

Close colocalization of CMTM7 and sIgM in B cells before and after sIgM ligation suggests a direct interaction of the two proteins, although we have not yet identified the region(s) in these proteins that are involved in this interaction. It was previously reported that the membrane-proximal C μ 4 domain of the μ H chain constant region is critical for the assembly of immobile sIgM oligomers that are competent for signaling when bound by monovalent, membrane-tethered antigens [27]. Although the mechanism for the C μ 4 domain-mediated sIgM oligomerization remains unknown, it may be mediated by integral membrane proteins that bind to this domain. It is tempting to speculate that CMTM7 binds to the C μ 4 domain of sIgM through its extracellular regions and thereby mediates oligomerization of the sIgM. We demonstrated that the CMTM7 ΔC mutant lacking the C-terminal extracellular region failed to associate with sIgM (**Figure 4C**), but the interpretation of this data is complicated by the fact that the ΔC variant is also not integrated into the plasma membrane. This might indicate that the C-terminal region of CMTM7 is necessary for the association with sIgM and this association is required for initial membrane integration of nascent CMTM7 at ER. This explanation would be unlikely, however, without a supposition that the C-terminal region can also be associated with other membrane receptor(s) expressed in HeLa cells (see Figure 3B). Alternatively, the C-terminal region may be intrinsically required for the membrane integration of CMTM7, although there has been no evidence that a C-terminal region neighboring the last transmembrane domain is necessary for the membrane integration of multi-spanning proteins. If the latter is the case, the role of the C-terminal region of CMTM7 in sIgM association remains obscure. Thus, further study is necessary to determine the molecular basis for the interaction of CMTM7 and sIgM.

Our confocal microscopy analysis indicated that CMTM7 is also associated with clathrin (**Figure 1A**) and co-internalized with sIgM after its ligation, although the majority of the sIgM-CMTM7 complex appears to remain on the cell surface at least for ten minutes after sIgM ligation before it is internalized (**Figure 1C**). Therefore, CMTM7 may also play some role in the process of clathrin-mediated sIgM internalization, but well after the early phase of BCR-mediated signal transduction. Concerning its role in ligation-induced sIgM internalization, we observed that this process was delayed in BAL17 cells overexpressing CMTM7 and, conversely, was accelerated in the CMTM7-knockdown BAL17 cells. Normal kinetics could by restored by reconstitution of the knockdown cells with full or ΔN, but not ΔC, forms of CMTM7 (our unpublished data). Thus, it appears that CMTM7, perhaps with associated BLNK, negatively regulates sIgM internalization to retain the BLNK-nucleated signalosome at the plasma membrane for the requisite time period required for optimal signal transduction. After that point, sIgM is presumably dissociated from Igα/β, which remains at the plasma membrane [28], [29], CMTM7 dissociates from BLNK, which is now bound to Igα, and then the sIgM, accompanied by CMTM7, may be endocytosed via clathrin-coated pits and vesicles and delivered to early endosomes.

We previously identified CMTM3, another member of the CMTM protein family, as a binding partner of BLNK, and showed that it binds to an N-terminal part of BLNK [23]. We observed that the expression level of *CMTM3* mRNA is relatively low compared to that of *CMTM7* in BAL17 cells, while it is relatively high in the DC2.4 dendritic cell line (our unpublished data). Indeed, according to the expression profiles in the RefDIC public database (http://refdic.rcai.riken.jp/welcome.cgi), *CMTM7* is highly expressed in almost all hematopoietic lineage cells, but high expression of *CMTM3* is restricted to dendritic cells and macrophages. Therefore, CMTM7 appears to play a more dominant role in B cells than CMTM3. On the other hand, CMTM3 may be more important in dendritic cells and macrophages, in which BLNK as well as an analogous adaptor protein SLP76 are expressed. In this regard, we found that CMTM3, but not CMTM7, binds also to SLP76 in DC2.4 cells and Raw263.7 macrophage cells (our unpublished data). We have observed distinct functions of BLNK and SLP76 in endocytosis and signaling through a cell-surface receptor in dendritic cells (manuscript in preparation), a finding that might be related to the different binding preferences of these adaptor molecules for CMTM3 and CMTM7.

## Materials and Methods

### Antibodies

The following antibodies were used: mouse anti-GM130, HRP-conjugated mouse anti-phosphotyrosine (PY20) (BD Biosciences); goat anti-EEA1 (C-15), goat anti-Btk (C-20), rabbit anti-ERK2 (C-14), rabbit anti-JNK1 (C-17), rabbit anti-Syk (N-19), rabbit anti-PLCγ2 (Q-20), rabbit anti-Lyn (44), FITC-conjugated donkey anti-goat IgG (Santa Cruz Biotechnology); rabbit anti-phospho-p44/42 MAPK (Thr202/Try204), rabbit anti-phospho-PLCγ2 (Tyr1217), rabbit anti-phospho-SAPK/JNK (Tyr183/Tyr185), rabbit anti-phospho-ZAP70/Syk (Tyr319/352), rabbit anti-phospho-Src (Tyr416) (Cell Signaling Technology); mouse anti-calnexin, rabbit anti-T7 (Chemicon); mouse anti-FlagM2 (Sigma-Aldrich); mouse anti-clathrin (Abcam); mouse anti-T7 (Novagen); goat anti-HA (Bethyl Laboratories); goat F(ab′)_2_ anti-mouse IgM, TRITC-conjugated donkey anti-rabbit IgG, HRP-conjugated goat anti-rabbit IgG (Jackson ImmunoResearch); biotin-goat F(ab′)_2_ anti-mouse IgM, FITC-conjugated goat anti-mouse IgG, HRP-conjugated goat anti-mouse IgM (Southern Biotech); goat anti-mouse IgM (Cappel); HRP-conjugated rabbit anti-mouse IgG (Zymed); FITC-conjugated streptavidin (Biolegend); rabbit anti-BLNK (Hayashi et al., 2000).

### Cell culture

BAL17 [30], HeLa [31] and HEK293T [32] cells were obtained from Dr. Takeshi Watanabe (Graduate School of Medicine, Kyoto University). BAL17 cells and their derivatives were incubated in RPMI1640 medium, and HeLa and HEK293T cells in DMEM, each supplemented with 10% FBS (Biological Industries) and penicillin/streptomycin. RPMI1640 medium also contained 50 mM 2-mercaptoethanol. For B-cell stimulation, B cells were treated with 10 µg/ml of goat F(ab′)_2_ anti-mouse IgM, unless otherwise noted.

### Real time RT-PCR

cDNA was synthesized with ReverTra Ace (Toyobo). Real-time PCR was performed with an Applied Biosystems 7500Fast. To amplify *CMTM7* cDNA, the following primers were used: 5′agatggtcaccctgctgatt3′ and 5′caggtgagcacacggtagaa3′.

### Plasmid constructions

The human CMTM7 (hCMTM7), mouse CMTM7 (mCMTM7) and its variants (ΔN; 38–168 aa, ΔC; 1–152 aa) were cloned into the pCAT7 vector [23]. The mCMTM7 and those appended with an N-terminal HA-tag or a C-terminal 3×FLAG-tag via a glycine/serine linker (GGGGS) were also cloned into a pcDNA3.1 vector. To generate *CMTM7*-knockdown cells (kd1 and kd2), short hairpin (shRNA)-expressing retroviral vectors were constructed using the pSIREN-RetroQ vector (Clontech) and the following RNA interference sequences: for *mCMTM7*, 5′acccagtcttcagatgcatct3′ (shRNA-1 for kd1) and 5′gccttcatctgtgtacgaagc3′ (shRNA-2 for kd2); and for an irrelevant sequence (*luciferase*) used for mock-transduction, 5′gtgcgttgctagtaccaac3′. To reconstitute the kd1 cells, the *mCMTM7* sequence was mutated at the shRNA-1 targeting site by PCR using two complementary primers, 5′cctgtataacccagtc**g**tc**g**gatgcatctgcctg3′ and 5′caggcagatgcatc**c**ga**c**gactgggttatacagg3′ (mutated nucleotides are indicated by bold letters), pCAT7-mCMTM7 and pCAT7-mCMTM7 ΔN as templates, and KOD polymerase (Toyobo). PCR products were treated with Dpn1 to digest the template DNA and used for bacterial transformation. The CMTM7 sequences of the resultant clones were verified by nucleotide sequencing.

### Transfection

HeLa cells were transfected with a pCAT7-based plasmid by Trans IT-LT1 (Invitrogen), and the cells expressing hCMTM7 were bulk selected with G418 (10 µg/ml) as indicated. BAL17 cells were transfected by electroporation (250 V, 950 µF) with pCAT7-based plasmids and clonally selected with G418 (1 µg/ml). The shRNA-expressing retroviral vectors were transduced as described [33] into BAL17 cells, which were then bulk selected with puromycin (20 µg/ml). HEK293T cells were transiently transfected with pcDNA3.1-based plasmids by Hily Max (Dojindo).

### Immunoprecipitation and Western blot analysis

Immunoprecipitations were performed as described [19]. The immunoprecipitated proteins and/or cell lysates were resolved by SDS-PAGE, transferred onto polyvinylidene fluoride membranes, probed with the indicated horseradish peroxidase (HRP)-labeled or unlabeled antibodies, and with the secondary HRP antibodies for the latter, and the signals were visualized with Western Lightning Chemiluminescence Reagent Plus (PerkinElmer). The blot was stripped off and reprobed with other antibodies as indicated. The chemiluminescence of the protein bands was detected and imaged by LAS-3000 (Fujifilm), the intensities of which were quantified using the Science Lab 2001 Image Gauge software (Fujifilm). The values of the intensities of phosphorylated proteins were normalized as relative to those of the corresponding total proteins, and expressed as relative values in each data.

### Immunofluorescence microscopy

HeLa cell transfectants cultured on glass-bottomed dishes were fixed with 4% paraformaldehyde, permeabilized with 0.1% TrironX-100, then stained with rabbit anti-T7 and the other indicated antibodies, and then with TRITC-anti-rabbit IgG antibody and appropriate FITC-labeled secondary antibodies. BAL17 cell transfectants were stained with biotin-goat F(ab′)_2_ anti-mouse IgM and rabbit anti-T7 antibodies on ice, incubated at 37°C for the indicated time periods, or kept on ice (for 0 min), then fixed with 4% paraformaldehyde and permeabilized with 0.1% Tween-100, and stained with anti-FcγRII/III blocking Ab (2.4G2). The cells were then incubated with blocking buffer (5% skim milk in PBS), stained with TRITC-anti-rabbit IgG antibody and FITC-streptavidin, extensively washed, and finally placed on slide glasses and coverslipped. The samples were analyzed with a Leica TCS SP2 confocal laser-scanning microscope (CLSM) using a 63× objective.

### Subcellular fractionation

For preparation of subcellular fractions, cells were suspended in hypotonic solution (20 mM Tris, 5 mM EDTA, 5 mM EGTA) for 10 min, passed through a 26-gauge needle for 15 strokes on ice, and centrifuged at 10,000× g for 5 min at 4°C. The supernatant fraction was further centrifuged at 110,000× g for 15 min at 4°C. The pellet and supernatant were used as membrane and cytoplasmic fractions, respectively. The membrane fraction was solubilized with 1% TNE buffer containing 1% NP-40 [19].

All the data shown are representative of three or more independent experiments

## Supporting Information

Figure S1 **CMTM7 is required for BLNK association with Syk, its phosphorylation and signal transduction from BCR.** (A, B, C, D) The CMTM7-knockdown (kd2) or mock-transduced BAL17 cells were stimulated with anti-IgM antibody for the indicated time periods and then analyzed as in Figure 2 (B, C, D, E).
(TIF)
