# Supplementary figures and images for: Identification of CMTM7 as a Transmembrane Linker of BLNK and the B-Cell Receptor
Source: PLoS One. 2012 Feb 21;7(2):e31829. doi: 10.1371/journal.pone.0031829 (PMC3283690; doi:10.1371/journal.pone.0031829)

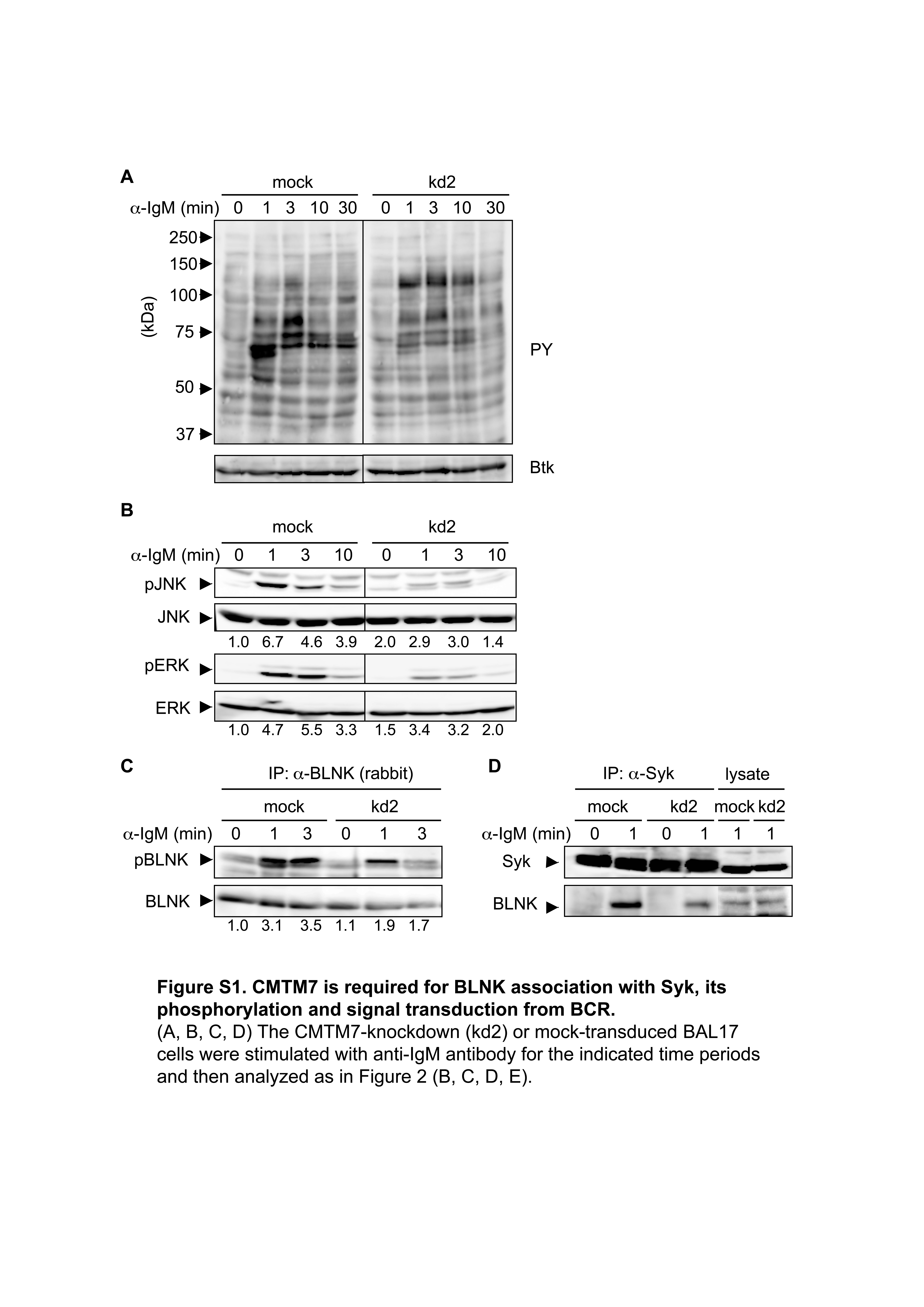

Supplement: Figure S1 — CMTM7 is required for BLNK association with Syk, its phosphorylation and signal transduction from BCR. (A, B, C, D) The CMTM7-knockdown (kd2) or mock-transduced BAL17 cells were stimulated with anti-IgM antibody for the indicated time periods and then analyzed as in Figure 2 (B, C, D, E). (TIF) [file pone.0031829.s001.tif]
